# Subdivision of arthropod *cap-n-collar* expression domains is restricted to Mandibulata

**DOI:** 10.1186/2041-9139-5-3

**Published:** 2014-01-09

**Authors:** Prashant P Sharma, Tripti Gupta, Evelyn E Schwager, Ward C Wheeler, Cassandra G Extavour

**Affiliations:** 1 Division of Invertebrate Zoology, American Museum of Natural History, Central Park West at 79th Street, New York, NY 10024, USA; 2 Department of Organismic and Evolutionary Biology, Harvard University, 26 Oxford Street, Cambridge, MA 02138, USA

**Keywords:** Amphipod, *cap-n-collar*, *Centruroides*, *Deformed*, Harvestman, Labrum, Mandible, *Parhyale*, *Phalangium*, Scorpion

## Abstract

**Background:**

The monophyly of Mandibulata - the division of arthropods uniting pancrustaceans and myriapods - is consistent with several morphological characters, such as the presence of sensory appendages called antennae and the eponymous biting appendage, the mandible. Functional studies have demonstrated that the patterning of the mandible requires the activity of the Hox gene *Deformed* and the transcription factor *cap-n-collar* (*cnc*) in at least two holometabolous insects: the fruit fly *Drosophila melanogaster* and the beetle *Tribolium castaneum*. Expression patterns of *cnc* from two non-holometabolous insects and a millipede have suggested conservation of the labral and mandibular domains within Mandibulata. However, the activity of *cnc* is unknown in crustaceans and chelicerates, precluding understanding of a complete scenario for the evolution of patterning of this appendage within arthropods. To redress these lacunae, here we investigate the gene expression of the ortholog of *cnc* in *Parhyale hawaiensis*, a malacostracan crustacean, and two chelicerates: the harvestman *Phalangium opilio*, and the scorpion *Centruroides sculpturatus*.

**Results:**

In the crustacean *P. hawaiensis*, the segmental expression of *Ph-cnc* is the same as that reported previously in hexapods and myriapods, with two distinct head domains in the labrum and the mandibular segment. In contrast, *Po-cnc* and *Cs-cnc* expression is not enriched in the labrum of either chelicerate, but instead is expressed at comparable levels in all appendages. In further contrast to mandibulate orthologs, the expression domain of *Po-cnc* posterior to the labrum is not confined within the expression domain of *Po-Dfd*.

**Conclusions:**

Expression data from two chelicerate outgroup taxa suggest that the signature two-domain head expression pattern of *cnc* evolved at the base of Mandibulata. The observation of the archetypal labral and mandibular segment domains in a crustacean exemplar supports the synapomorphic nature of mandibulate *cnc* expression. The broader expression of *Po-cnc* with respect to *Po-Dfd* in chelicerates further suggests that the regulation of *cnc* by *Dfd* was also acquired at the base of Mandibulata. To test this hypothesis, future studies examining panarthropod *cnc* evolution should investigate expression of the *cnc* ortholog in arthropod outgroups, such as Onychophora and Tardigrada.

## Background

### Gene expression as evidence for phylogenetic relationships

As indicators of phylogenetic relationships, arthropod embryonic gene expression patterns are among the most idiosyncratic, frequently lending themselves to ambiguous statements of homology. This stems in part from limitations in taxonomic sampling; comparative gene expression data are presently available from approximately 25 arthropod species [1-4], a minuscule fraction of those for which nucleotide sequence data have been collected. In addition, as evolutionary developmental biology is often driven by inquiry into the origins of particular morphological structures, the state of a gene’s deployment is often not assessed in lineages and/or specific stages that lack a structure of interest, thereby engendering gaps in comparable expression data. As a consequence, the degree to which expression patterns are conserved is largely unknown for many well-characterized genes involved in embryogenesis, barring such exceptions as anterior Hox genes, segmentation genes, limb-patterning genes, and some neurogenetic markers [1,5-7].

**Figure 1:**
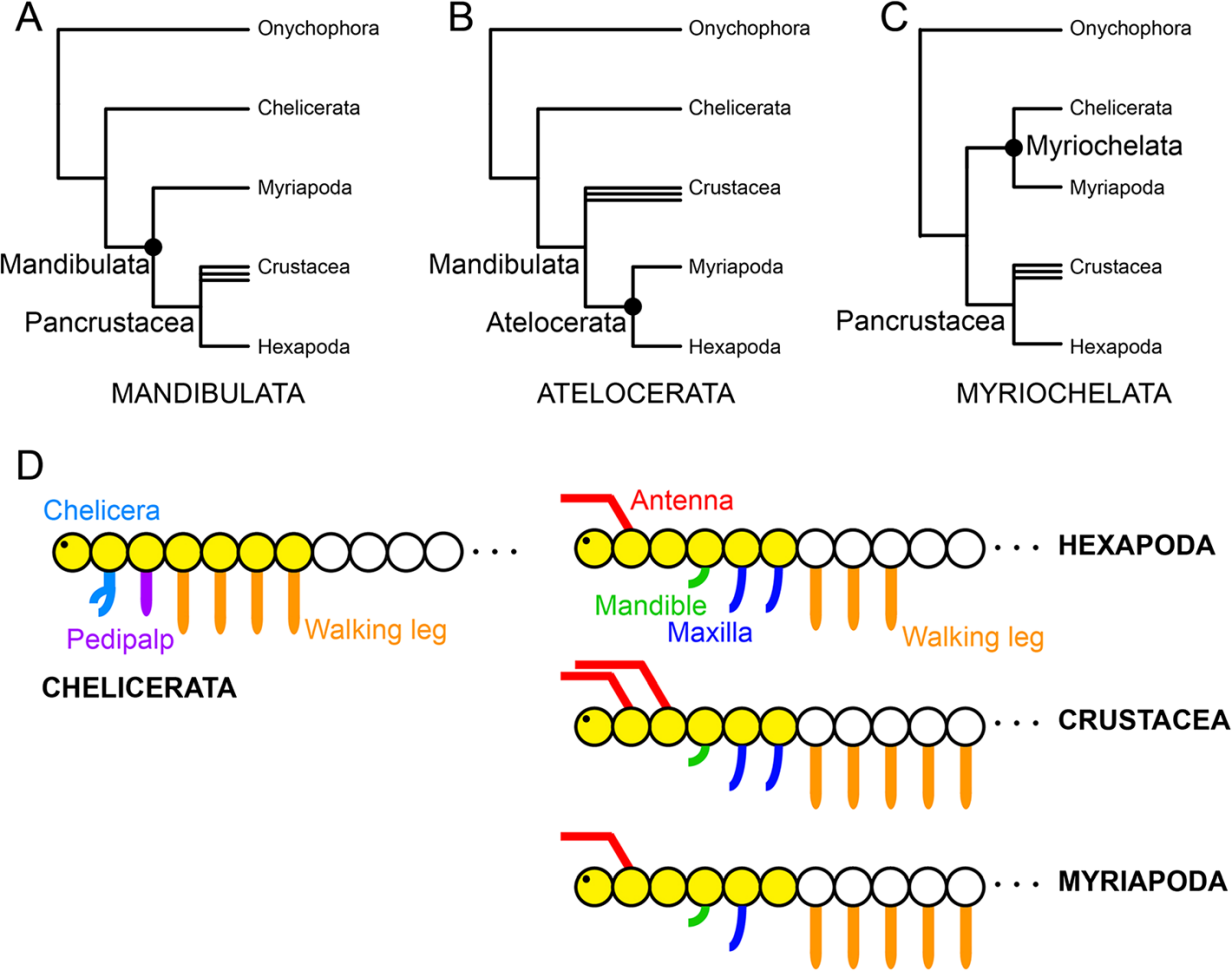
**Competing hypotheses in arthropod phylogeny. (A)** Mandibulata unites the non-chelicerate arthropods in a clade and is the hypothesis most stably recovered in phylogenetic analyses. Multiple terminal icons indicate the non-monophyly of Crustacea. **(B)** The Atelocerata hypothesis unites hexapods and myriapods to the exclusion of crustaceans. **(C)** Myriochelata unites the myriapods and chelicerates in a clade. **(D)** Segmental architecture of Arthropoda. The depiction of Myriapoda corresponds to the millipede bauplan (a second maxilla is present in other myriapod lineages). The anterior-most head segment, or protocerebral segment, is to the left. Yellow circles indicate the anterior tagmata, the chelicerate prosoma and the mandibulate head.

In other cases, even when broader taxonomic sampling increases confidence in assessments of the evolution of gene expression, the incidence of homoplasy (and particularly reversals) can create further ambiguity in the interpretation of evolutionary patterns. The sum of phylogenetic and phylogenomic studies of arthropods supports the monophyly of Mandibulata - a clade comprised of myriapods, a paraphyletic group commonly termed crustaceans, and hexapods - and its sister relationship to Chelicerata - a clade composed of arachnids, xiphosurans, and pycnogonids (Figure 1A) [8,9]. Morphological data are strongly consistent with these relationships: mandibulates are characterized by a six-segmented anterior tagma (the head) bearing antennae on the deutocerebral segment and no locomotory appendages, whereas the seven-segmented anterior tagma (the prosoma) of euchelicerates bears the namesake appendage on the deutocerebral segment, and all walking legs with a distinct podomere called the patella, which confers the appearance of a “double-knee”. Contrary to this topology, the head-patterning gene *collier* (*col*) is strongly expressed in the intercalary segment of myriapods and hexapods, but not in the corresponding segments of crustaceans (the second antennal segment), chelicerates (the pedipalpal segment), or onychophorans - the sister lineage of arthropods [10-12]. These data have been interpreted to mean a possible role for *col* in patterning the appendage-less tritocerebral state of hexapods [10]. Indeed, the perfect correspondence between the incidence of the intercalary segment and the expression domain of *col* is suggestive of a convergent function for *col* in patterning an appendage-less segment. However, an alternative interpretation of these data has been putative support for the Atelocerata hypothesis, which unites Myriapoda and Hexapoda as sister groups (Figure 1B) [11]. This interpretation is implicitly based on Dollo parsimony (non-reversibility of a given character state) and renders a large number of better-sampled morphological and molecular characters homoplastic [13]. A comparable example is the case of leg gap gene expression, where the expression boundaries of *homothorax* (*hth*) and *extradenticle* (*exd*) support the controversial sister relationship of Myriapoda and Chelicerata (Figure 1C) ( [7,14], but see [15]), a relationship otherwise only poorly supported by some analyses of molecular sequence data [16,17].

A counterexample of gene expression evolution consistent with the total evidence phylogenetic tree may be provided by examining an unambiguous synapomorphy of Mandibulata: the mandible. The eponymous biting appendage of mandibulates is a gnathobasic structure occurring on the fourth head segment of all mandibulates, irrespective of the architecture of the remaining head segments (Figure 1D) [18-20]. Two genes are required for the proper formation of the mandible: the Hox gene *Deformed* (*Dfd*) and the basic leucine zipper family transcription factor *cap-n-collar* (*cnc*) [21-23]. In the fruit fly *Drosophila melanogaster*, *Dfd* is required for patterning the mandibular and first maxillary segments [24]. In both *D. melanogaster* and the beetle *Tribolium castaneum*, *cnc* is expressed in two domains, the first in the labrum and the second in the mandibular segment [25]. Functional studies in both species have shown that *cnc* is required for formation of the labrum, and for differentiating the mandible from the maxilla. A loss-of-function mutation in *D. melanogaster* results in ectopic maxillary structures on the mandibular segment (for example, hooks and cirri), and RNA interference-mediated knockdown in *T. castaneum* in complete mandible-to-maxilla homeotic transformation [21-23,25]. In both insects, *cnc* downregulates the expression of *Dfd* in the mandibular segment over the course of mandibular limb bud growth. Intriguingly, *cnc* is activated by *Dfd* in *T. castaneum* but not in *D. melanogaster*[25]. The polarity of this regulation with respect to phylogeny is not known.

Expression data for orthologs of *cnc* are available for a hemimetabolous insect (*Oncopeltus fasciatus*), a non-metamorphic insect (*Thermobia domestica*) and a millipede (*Glomeris marginata*), all of which bear a characteristic labral and mandibular domain [12,26,27]. Intriguingly, the mandibular domains of all mandibulate *cnc* orthologs occur within the *Dfd* domains of these lineages, and a downregulation of *Dfd* in the mandibular segment of older stage embryos has been observed across mandibulates as well. These conserved expression dynamics have been used to suggest that the mandible-patterning function of *cnc* evolved at the base of Mandibulata within the domain of *Dfd*[25].

However, as demonstrated by the case of *col*, *hth*, and *exd*, many embryonic genes are prone to convergence and/or reversals. Inasmuch as *cnc* expression is unknown in crustaceans and non-mandibulate arthropods, the assignation of the two-domain head expression pattern to the ancestor of Mandibulata remains ambiguous. To refine the inference of evolution of *cnc* expression and its regulation by *Dfd*, we investigated the expression of *cnc* in the malacostracan crustacean *Parhyale hawaiensis* and two chelicerates: the harvestman *Phalangium opilio* and the scorpion *Centruroides sculpturatus*. We used these data to test the prediction that the two-domain head expression pattern is conserved in the crustacean, whereas an unknown, non-mandibulate state occurs in the chelicerates.

## Methods

### Embryo cultivation and fixation

*P. hawaiensis* adults were cultured in artificial seawater (Instant Ocean, Blacksburg, VA, USA) with crushed coral at 28°C. Animals were fed daily with ground aquaculture feed: 40% TetraPond® wheat germ sticks, 40% TetraMin® flake food, and 20% Tropical® spirulina (Tetra, Blacksburg, VA, USA). Gravid females were anesthetized with CO_2_, and embryos were collected as described previously [28]. Embryos were fixed for *in situ* hybridization by incubating in 3.7% formaldehyde in 1 × PBS for 2 minutes at 75°C followed by 20 minutes in 3.7% formaldehyde in 1 × PBS at 4°C. Membranes were manually dissected from embryos in PBS and embryos fixed overnight at 4°C.

Adults of the harvestman *P. opilio* were hand collected between 21.00 and 03.00 from Weston, Massachusetts, USA in May through July, 2013. Housing, feeding, embryo cultivation, and embryo fixation followed published protocols [29].

Adult females of the scorpion *C. sculpturatus* were purchased from an animal supplier (Hatari Invertebrates, AZ, USA). Females were anesthetized with CO_2_ and embryos dissected from the ovary following a modification of a published protocol [30]. Embryos were dissected to remove yolk and fixed in 3.7% formaldehyde in 1 × PBS at room temperature overnight.

### Gene identification and whole mount *in situ* hybridization

Potential orthologs of *cnc* were identified in the annotated developmental transcriptomes of *P. hawaiensis* (deposited in the ASGARD Project database; [31]), *P. opilio* (Sharma and Giribet, unpublished data), and *C. sculpturatus* (Sharma and Wheeler, unpublished data). For *C. sculpturatus*, an ortholog of the Hox gene *Antennapedia* was additionally identified and used as a positive control for the *cnc in situ* hybridization experiments. Gene identity of *cnc* orthologs was confirmed by BLAST and alignments generated from conceptual peptide translations (Additional file 1: Figure S1). Sequences of all genes are deposited in GenBank.

Templates for riboprobe synthesis for *P. hawaiensis* and *P. opilio* were generated following a published protocol [32]: genes were amplified by PCR using gene-specific primers (GSPs) with an added linker sequence (5′-ggccgcgg-3′ for the forward primer end and 5′-cccggggc-3′ for the reverse primer). A T7 polymerase binding site for anti-sense or sense probe synthesis was generated in a second PCR using the forward or reverse GSP and a universal primer binding to the 3′ or 5′ linker sequence with an added T7 binding site, respectively. GSPs were designed from the corresponding transcriptomic assemblies. For *P. opilio*, two pairs of sense and anti-sense probes with only partial overlap over the basic region leucine zipper domain were generated to establish the validity of the expression data. Templates for riboprobe synthesis for *C. vittatus* were generated by PCR-amplified GSPs, and cloning amplicons using the TOPO® TA Cloning® Kit with One Shot® Top10 chemically competent *Escherichia coli* (Invitrogen, Carlsbad, CA, USA), following the manufacturer’s protocol. Amplicon identities were verified by direct sequencing. A list of the GSPs used for generating sense and anti-sense probes is provided in Additional file 2: Table S1.

Whole mount *in situ* hybridization on *P. hawaiensis* embryos was performed as described previously [33] with the following modifications: prior to rehydration, embryos were cleared by incubation in xylene for 20 minutes. Hybridization was performed at 67°C. Following post-fixation, embryos were incubated in detergent solution (1.0% SDS, 0.5% Tween, 50.0 mM Tris–HCl (pH 7.5), 1.0 mM EDTA (pH 8.0), 150.0 mM NaCl) for 30 minutes and then fixed again in 3.7% formaldehyde for 30 minutes. After hybridization, embryos were washed twice in 2 × saline sodium citrate for 30 minutes and then twice in 0.2 × saline sodium citrate for 30 minutes. Probes were visualized using nitro-blue tetrazolium and 5-bromo-4-chloro-3'-indolyphosphate staining reactions, run overnight at 4°C.

*In situ* hybridization for *P. opilio* followed published protocols [29]. For *C. sculpturatus*, *in situ* hybridization followed the same protocol as for *P. opilio*. Staining reactions for detection of transcripts lasted between 0.5 and 6 hours at room temperature. Embryos were subsequently rinsed with 1 × PBS + Tween-20 0.1% to stop the reaction, counterstained with Hoechst 33342 (Sigma-Aldrich, St. Louis, MO, USA) 10 μg/ml to label nuclei, post-fixed in 4% formaldehyde, and stored at 4°C in glycerol. Embryos were mounted in glycerol and images were captured using an HrC AxioCam and a fluorescence zoom stereomicroscope driven by Zen (Zeiss, Oberkochen, Germany).

## Results

### Identification of *cnc* orthologs

**Figure 2:**
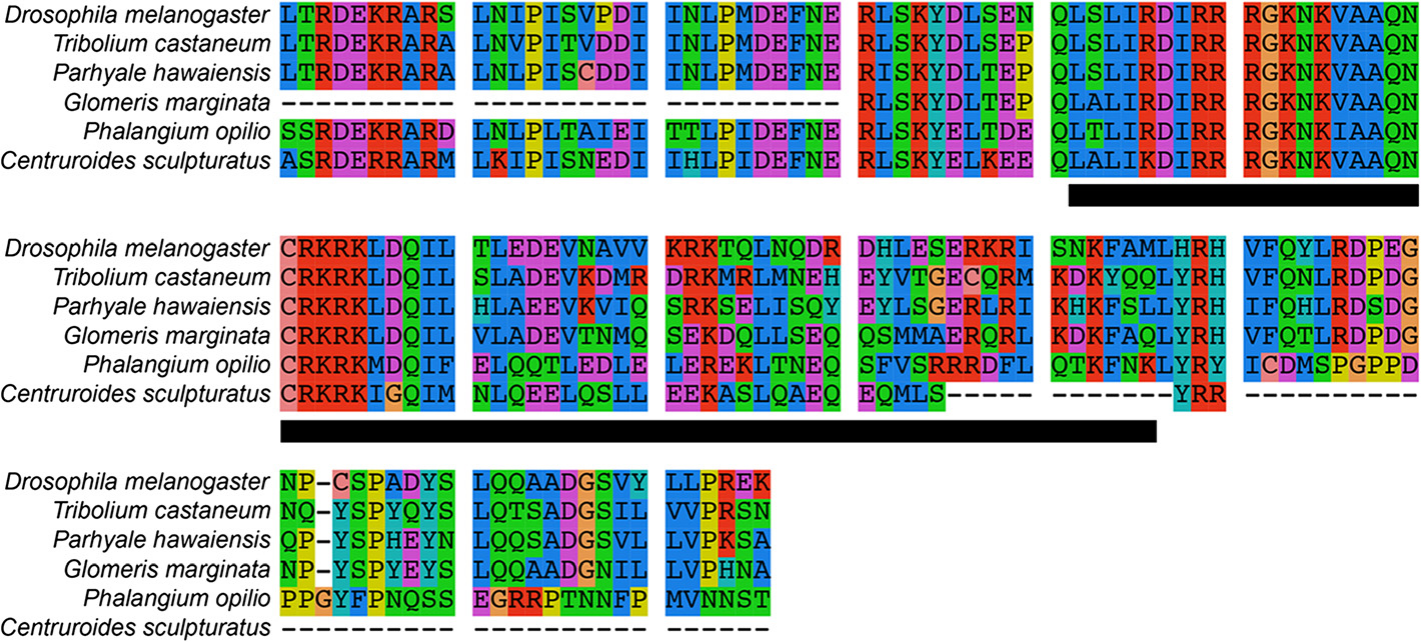
**Multiple sequence alignment of arthropod *cap-n-collar *orthologs.** The conserved region comprising 146 amino acids is shown. Black bar indicates the basic region leucine zipper domain.

Putative single-copy *cnc* orthologs between 393 bp and 739 bp in length were identified in the transcriptomes of all three species. To confirm gene orthology, multiple sequence alignment of *cnc* amino acid sequences was conducted using MUSCLE v. 3.6 [34], comparing crustacean and chelicerate sequences to those of *D. melanogaster*, *T. castaneum*, and *G. marginata*. The conserved region of the alignment is shown in Figure 2; the complete alignment is provided in Additional file 1: Figure S1. Next, we studied gene expression in the embryos of *P. hawaiensis* and both chelicerates. As negative controls, we tested for expression of sense probes. In all cases, no staining was observed in sense controls (Additional file 3: Figure S2).

### Expression of *cnc* in the crustacean *P. hawaiensis*

**Figure 3:**
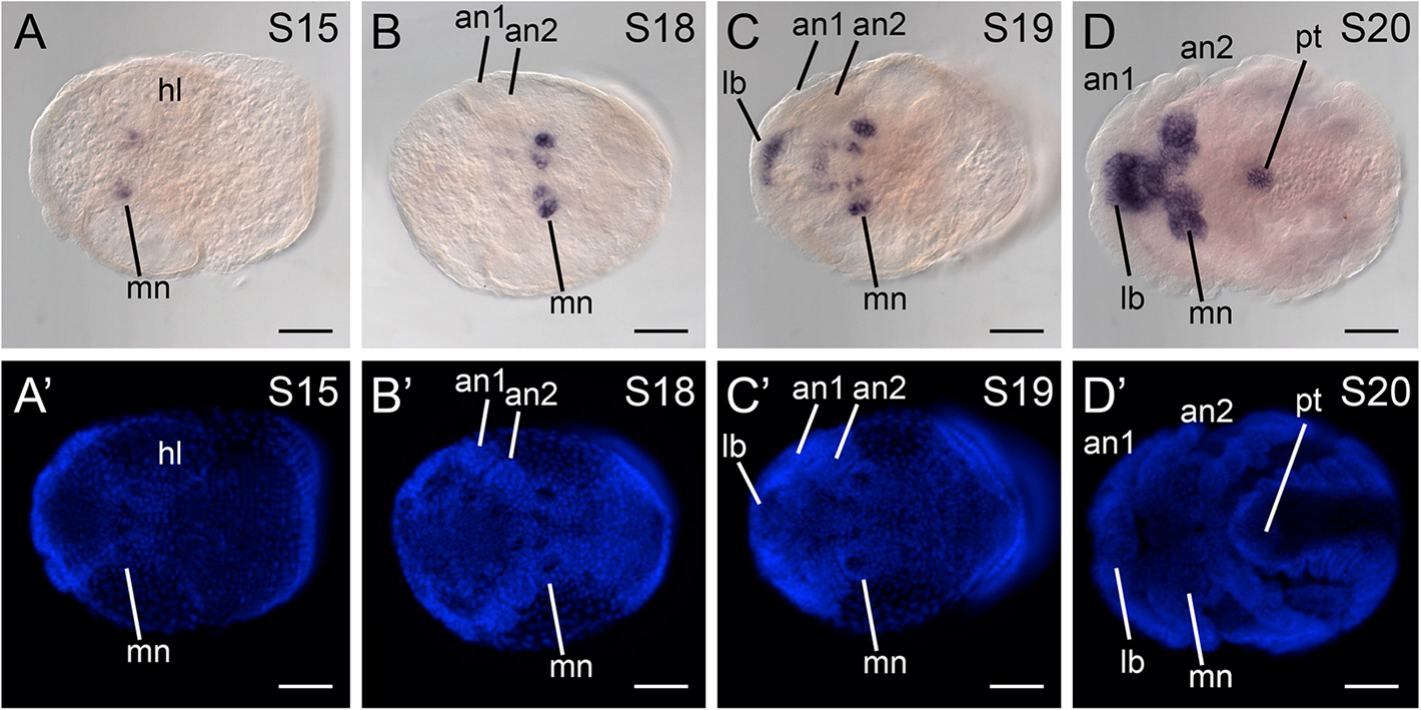
***Parhyale hawaiensis cap-n-collar *ortholog is expressed as in other mandibulates. (A)** Stage 15 embryo in ventral view. Expression of *Ph-cnc* is detected in the mandibular anlagen. **(B)** Stage 18 embryo in ventral view, showing expression of *Ph-cnc* in the mandibular limb buds. **(C)** Stage 19 embryo in ventral view. Expression of *Ph-cnc* occurs in discrete domains in the labrum, the mandibles, and in the ventral ectoderm of the mandibular segment. **(D)** Stage 20 embryo in ventral view. Expression of *Ph-cnc* encompasses the labrum, the stomodeal wall, the mandibles, and the proctodeum. **(A’-D’)** Counterstaining of embryo shown in **(A)** and **(D)** with Hoechst. an1, first antenna; an2, second antenna; lb, labrum; mn, mandible; pt, proctodeum. Scale bars for all figures are 100 μm.

Consistent with expression of *cnc* in other mandibulates, *Ph-cnc* is expressed in disjunct head domains in limb bud stage embryos (stages 18-22). Expression in earlier stages occurs in the mandibular segment (stages 15-18; Figure 3A,B), and expands into labrum and the stomodeal wall as the mandibles elongate (stages 19-20; Figure 3C,D). By stage 20, the anterior-most domain comprises strong expression in labrum and the tissues around the stomodeum, forming a ring. This domain does not extend into the head lobes. The mandibular domain consists of strong expression in the mandibular limb buds. By stage 20, an additional expression domain is observed at the posterior terminus of the embryo, in a ring around the proctodeum (Figure 3D).

### Expression of *cnc* in the harvestman *P. opilio*

**Figure 4:**
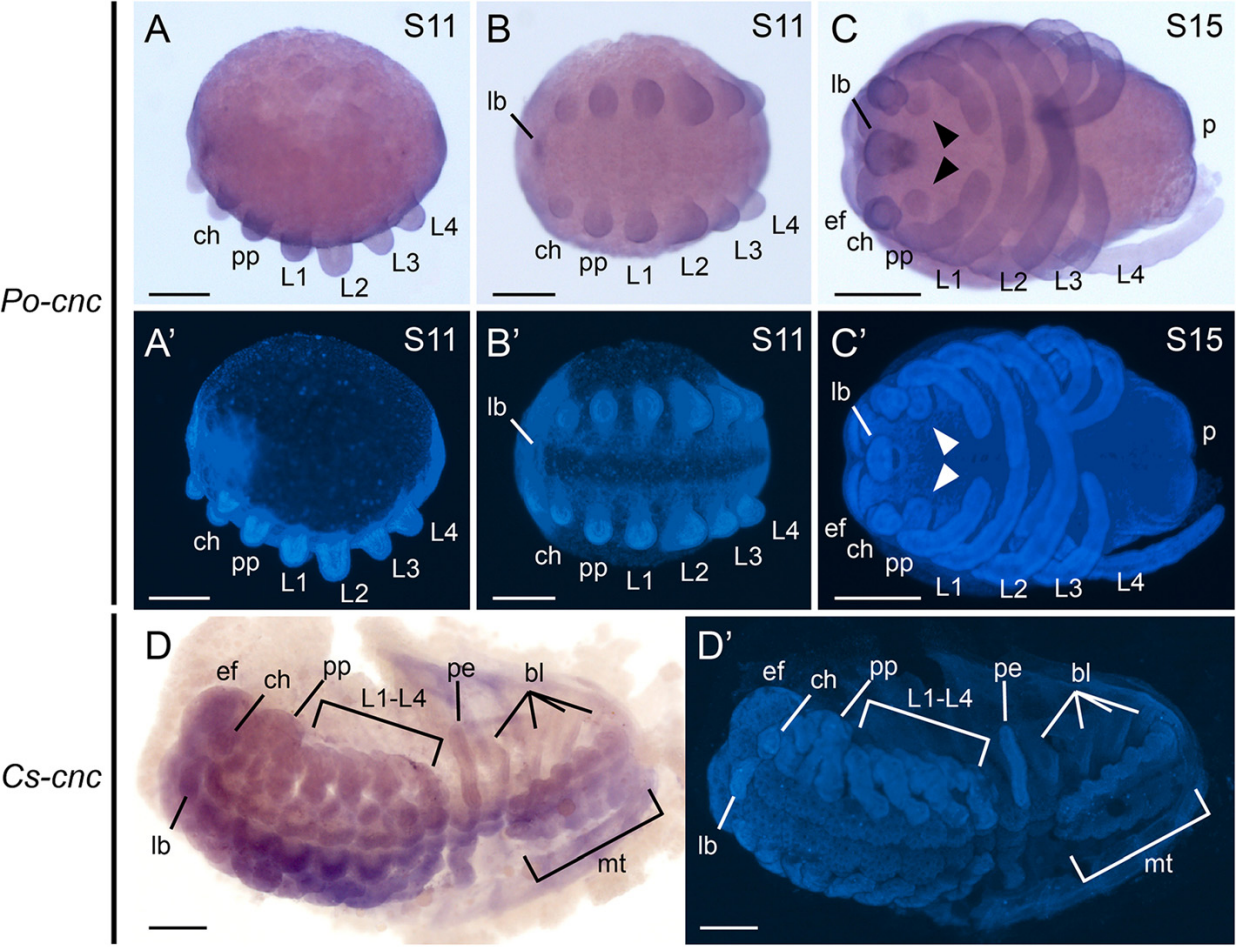
**Chelicerate *cap-n-collar *orthologs are expressed throughout the germ band. (A)** Stage 11 embryo of the harvestman *Phalangium opilio* in lateral view. *Po-cnc* (488-bp anti-sense probe) is expressed throughout the germ band, including in all prosomal appendages. **(B)** Same embryo as in **(A)** in ventral view. **(C)** Stage 15 embryo of *P. opilio* in ventral view. *Po-cnc* continues to be expressed throughout the germ band. Expression is additionally detected in the coxapophyses (arrowheads). **(D)** Expression of *Cs-cnc* in the scorpion embryo. *Cs-cnc* is detected throughout the germ band, including in the eye fields, all prosomal and opisthosomal appendages, and in the metasoma (tail). **(A’-D’)** Counterstaining of embryos shown in **(A-D)** with Hoechst. bl, book lung; ch, chelicera; ef, eye field; lb, labrum; L1-L4, leg 1-leg 4; mt, metasoma; p, posterior end; pe, pectine; pp, pedipalp. Scale bars are 200 μm for **(A-C)** and 500 μm for **(D)**.

In early stages (stage 11), *Po-cnc* is expressed in the labrum, as well as all limb buds and the posterior end (Figure 4A,B). No expression is observed along the ventral midline of the prosomal segments. In older stages (stage 15), *Po-cnc* continues to be expressed in the labrum and all prosomal appendages, as well as outgrowing endites of the pedipalpal and L1 segments (Figure 4C). Comparably to *P. hawaiensis*, the labral domain extends into a ring of expression surrounding the stomodeum. Expression is observed in the eye fields as well as the posterior terminus. Identical expression patterns were obtained with either of two partially overlapping anti-sense probes, and no expression was observed with either complementary sense probe (Additional file 3: Figure S2; Additional file 4: Figure S3A-C).

### Expression of *Antp* and *cnc* in the scorpion *C. sculpturatus*

There are currently no well-established laboratory scorpion model species, and due to the peculiar life history traits of scorpions (including live birth, small broods, gestation periods lasting multiple months), collecting embryos is largely a matter of chance. Obtaining specific developmental stages is thus a matter of intensive sampling of adult females. In the present study, we obtained embryonic stages of *C. sculpturatus* comparable to stage 15 of *P. opilio* (Figure 4C,D), as inferred from (1) completion of appendage podomerization, (2) formation of gnathobases, and (3) completion of opisthosomal segment addition.

In order to establish the validity of the *in situ* hybridization protocol for this species, gene expression of the *Antp* ortholog was additionally investigated. We reasoned that the conservation of the *Antp* expression domain in multiple chelicerate species [1,29] would make this gene an appropriate choice as a positive control. Using a *Cs-Antp* anti-sense probe, we found that, as in all known chelicerates, the anterior expression boundary of *Cs-Antp* occurs in the posterior part of the L4 segment. *Cs-Antp* is expressed throughout the posterior tagmata (mesosoma and metasoma). Complete absence of staining is observed in the prosoma (Additional file 4: Figure S3D) and in sense controls (not shown), suggesting that our *in situ* protocol can effectively distinguish signal from background.

Using this *in situ* hybridization protocol, we found that *Cs-cnc* is expressed throughout the prosoma, including in the eye fields, the labrum, the appendages, the coxapophyses, and the ventral ectoderm (Figure 4D). *Cs-cnc* is additionally expressed in the mesosomal ventral ectoderm, the pectines, the book lungs, and throughout the metasoma. Expression is weakest in the periphery of the O4-O7 segments, which bear the book lungs.

## Discussion

Beyond testing a particular evolutionary scenario through repeated observation of a putatively conserved trait, extensive sampling of lineages for a character of interest is essential for identifying the origins of evolutionary novelties, such as the arthropod mandible. Here we investigated the evolution of *cnc* expression and tested the association of *cnc* domains with mandibular patterning. Given that published expression data are available only for four insects and one myriapod [21-27], we aimed to corroborate the conservation of *cnc* domains for the first time in a crustacean species, and infer their origin by examining *cnc* expression in the sister group of mandibulates, the chelicerates.

### *Ph-cnc* expression supports the archetypal mandibulate pattern

The localization of *Ph-cnc* transcripts in the labrum and mandibular segments of the malacostracan *P. hawaiensis* - the “cap” and “collar” domains, respectively - supports this characteristic expression pattern as conserved among mandibulates. The restriction of the posterior head domain within the mandibular segment, in concert with the known function of *cnc* in patterning mandibular identity in both *D. melanogaster* and *T. castaneum*, suggests conservation of *cnc* function among mandibulates with respect to mandibular patterning. Similarly, conserved expression of *cnc* in the labrum of all sampled mandibulates suggests that *cnc* is required for the development of this structure; in *D. melanogaster* and *T. castaneum*, loss-of-function of *cnc* results in the deletion of the labrum [21,25].

One *cnc* expression domain of unknown function in mandibulates is expression in the posterior-most segments. In *P. hawaiensis*, *Ph-cnc* is expressed in a ring of tissue surrounding the proctodeum (Figure 3D). Such a posterior expression domain occurs variably among insects; in *D. melanogaster*, *T. castaneum*, and *O. fasciatus*, *cnc* is not expressed in the posterior-most segments [21,25,27], whereas in the firebrat *T. domesticus*, *Td-cnc* is expressed from the A6 segment to the posterior terminus [26]. The functional significance of the posterior domain is not known, but may represent an evolutionary remnant of the unrestricted *cnc* domain in the non-mandibulate arthropods.

### Chelicerate ortholog expression suggests subdivision of *cnc* domains in the mandibulate ancestor

**Figure 5:**
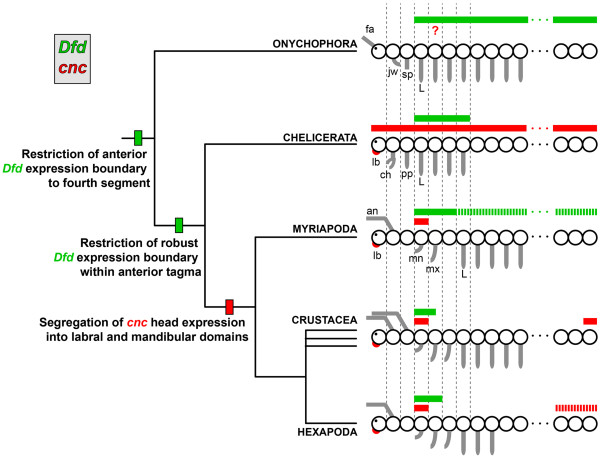
**Inferred evolution of *cap-n-collar *and *Deformed *in Arthropoda.** Known expression patterns of *cnc* (red) and *Dfd* (green) from panarthropods suggest restriction of a robust *Dfd* domain to within an anterior tagma in the ancestor of Arthropoda. Hashed bars for myriapods indicate weak expression of *Dfd* in the trunk segments of the millipede (but not the centipede). Whereas *cnc* expression occurs throughout the developing chelicerate embryo, disjunct domains of *cnc* expression are exclusive to the mandibulates, thereby constituting a putative mandibulate synapomorphy. Hashed bars in the posterior terminus for hexapods indicate presence of *cnc* in the posterior segments of some insects. ch, chelicera; fa, frontal appendage; jw, jaw; sp, slime papillae; L, leg; lb, labrum; mn, mandible; mx, maxilla.

The conservation of the disjunct head expression domains of *cnc* among the mandibulates precludes assessment of their evolutionary origin based on mandibulate data alone. To assess the evolution of the genetic network that may have precipitated the patterning of the mandible, we examined *cnc* expression in two chelicerates, the harvestman and the scorpion. As inferred from Hox gene data, specifically the anterior boundary of *Dfd*, the mandibular segment corresponds to the first walking leg segment in chelicerates (Figure 5) [1]. Given that more anterior appendages are used for feeding in Chelicerata, we hypothesized that *cnc* chelicerate expression would occur as a non-mandibulate but unknown state.

Consistent with this hypothesis, gene expression of *cnc* orthologs in both the harvestman and the scorpion indicate nearly ubiquitous expression in examined developmental stages (Figure 4C,D; compare to Figure 3). In the early stages sampled for *P. opilio*, expression is observed throughout the germ band (Figure 4A,B). *Po-cnc* continues to be ubiquitously expressed throughout both the prosoma and opisthosoma at the developmental stage when the appendages are fully podomerized and elongate (stage 15) (Figure 4C). While we were unable to examine early limb bud stages of scorpions (prior to completion of opisthosomal segmentation), we observed a similar expression pattern in scorpion developmental stages with fully podomerized appendages and morphologically distinct opisthosomal organs (pectines and book lungs) (Figure 4D).

The function of *cnc* in Chelicerata was not examined here, due to the lack of functional tools in the scorpion and the limited seasonality of the harvestman. Beyond arthropods, the functions of orthologs of *cnc* have been studied in the nematode *Caenorhabditis elegans* and in vertebrates. In *C. elegans*, the ortholog of *cnc*, *skn-1*, is required for the specification of ventral blastomere identity at the four-cell stage. In *skn-1* mutants the EMS blastomere, which normally forms pharyngeal and intestinal cells, acquires P2 cell identity and forms body wall muscle and hypodermal cells [35]. *Nrf2*, a vertebrate *cnc* ortholog, has been implicated in oxidative stress response in mammals [36,37], a non-developmental role similarly observed in xenobiotic response in *Drosophila*[38]. These disparate functional data are suggestive of multiple co-options of *cnc* throughout Bilateria to achieve various functions and preclude speculation on the role of *cnc* in chelicerates.

The ubiquitous expression of chelicerate *cnc* expression suggests that the expression and function of *cnc* in distinct head appendage domains is exclusive to Mandibulata and presumably evolved in the ancestor of mandibulates. Alternatively, an equally parsimonious reconstruction could be evolution of subdivided *cnc* domain at the base of Panarthropoda, and subsequent secondary evolution of the chelicerate state of *cnc* expression. Under this hypothetical scenario, the *cnc* ortholog of Onychophora would be predicted to have an expression domain comparable to that of Mandibulata.

However, we consider a shared expression pattern in Mandibulata and Onychophora unlikely for several reasons. First, like Chelicerata, Onychophora lack a mandible. Second, the first walking leg segments of both onychophorans and chelicerates are putatively homologous to each other, and to the mandibular segment of Mandibulata; only the first three head segments of Onychophora and Chelicerata have identities distinct from the walking legs, in contrast to the six-segmented mandibulate head (Figure 5). For these reasons, we consider a shared state between Onychophora and Chelicerata plausible. Nevertheless, assignation of *cnc* subdivision to the base of Mandibulata remains ambiguous, and it is imperative to investigate *cnc* expression in onychophorans and tardigrades to test this putative synapomorphy of mandibulates in future studies.

### Regulation of *Dfd* by *cnc* may have evolved within Mandibulata

In all presently sampled branches of the mandibulate tree (hexapods, malacostracan crustaceans, and myriapods), part of *cnc* expression is restricted to within the *Dfd* expression domain. In hexapods, the expression domain of *Dfd* spans the mandibular and maxillary segments (Figure 5). *cnc* arises within the *Dfd* domain and progressively downregulates *Dfd*, with declining levels of *Dfd* expression signal in the mandibular segment over time [25]. Intriguingly, the temporal expression of *Dfd* follows the same pattern in the millipede *G. marginata*, with loss of expression in the distal mandible in older stages (Figure 4A-C of [39]). A similar expression pattern has been reported in the mandibular segment of the centipede *Lithobius atkinsoni*, namely the absence of *Dfd* expression in the distal mandible (note that the posterior boundary of *Dfd* is not the same in the two species; weak expression of *Dfd* is observed in the millipede trunk, but not in the centipede) (Figure 4C of [40]). These observations suggest conservation of the regulation of *Dfd* by *cnc* in the mandibular segment of non-hexapod mandibulates. Unfortunately, functional tools are currently lacking in myriapods, precluding a direct test of this genetic interaction in either centipedes or millipedes.

In contrast to mandibulates, known *Dfd* expression in euchelicerates with eight-legged embryos (that is, all chelicerates except Pycnogonida, Acariformes, Parasitiformes, and Ricinulei) is restricted to the four walking leg segments, and does not wane in expression strength in the course of development [29,41,42]. Moreover, the occurrence of *cnc* transcripts throughout the embryo, rather than exclusively within the chelicerate *Dfd* domain, disfavors regulation of *cnc* by *Dfd* in a manner comparable to the mandibulates’ regulatory apparatus (Figure 5). These data suggest that the downregulation of *Dfd* within a specific *cnc* domain constitutes a synapomorphy of Mandibulata that is required for the patterning of the mandible.

The interrelated evolution of *cnc* and *Dfd* may be investigated in future by characterizing the expression domain of *cnc* in onychophorans. Previous description of onychophoran Hox gene expression domains has reported broad expression of *labial* (*lb*), *proboscipedia* (*pb*), *Hox3*, and *Dfd* transcripts, from anterior boundaries shared with arthropods extending to the posterior terminus of the velvet worm embryo [43]. It has previously been suggested that the restriction of the posterior expression boundaries of Hox genes in arthropods precipitated the evolution of various tagmata (Figure 5) [1,43]. Ubiquitous expression of onychophoran *cnc*, comparable to expression of *cnc* orthologs in chelicerates, would lend support to the evolutionary inferences made herein.

We suggest that future studies endeavoring to investigate mandible evolution should focus on two avenues of research: (1) developing functional tools in a species of Myriapoda to interrogate the regulatory dynamic of *cnc* and *Dfd* in a basally branching mandibulate, and (2) identifying the function of *cnc* in chelicerates. While several aforementioned aspects of scorpion life history will delay the development of functional tools in *C. sculpturatus*, RNA interference has proven successful in spiders, mites and, most recently, harvestmen [44-46].

## Conclusion

The evolution of the mandible, an arthropod evolutionary novelty, has previously been linked to the function of *cnc*, and conserved expression of *cnc* orthologs was heretofore observed in insects and a millipede. Here we investigated the expression of *cnc* in a malacostracan crustacean and two chelicerates. We show that *cnc* expression is conserved in all branches of the mandibulate phylogeny. By contrast, chelicerate *cnc* is ubiquitously expressed in examined developmental stages, suggesting that evolution of the mandible may have involved the progressive subdivision of the *cnc* expression domain.

## Abbreviations

BLAST: Basic local alignment search tool; bp: Base pair; GSP: Gene-specific primers; PBS: Phosphate buffered saline; PCR: Polymerase chain reaction

## Competing interests

The authors declare that they have no competing interests.

## Authors’ contributions

PPS conceived of the study. PPS and WCW generated the scorpion developmental transcriptome. CGE generated the crustacean developmental transcriptome and provided funding for part of the study. TG collected and analyzed the crustacean data; EES and PPS collected chelicerate data. EES, PPS, and TG analyzed expression patterns and drafted the manuscript. All authors edited the manuscript and approved the final content for submission.

## Supplementary Material

Additional file 1: Figure S1. Complete multiple sequence alignment of *cap-n-collar* orthologs. Click here for file

Additional file 2: Table S1. Gene-specific primers used for synthesizing *cap-n-collar* probes. Click here for file

Additional file 3: Figure S2. Chelicerate *cap-n-collar* sense probes. (A) Stage 13 embryo of *Phalangium opilio* stained with 488-bp sense probe in same solution and for the same period of time as embryos shown in Figure 4A-C. (B) Stage 14 embryo of *Phalangium opilio* stained with 739-bp sense probe in same solution and for the same period of time as embryos shown in Additional file 4: Figure S3B-C. (C) *Centruroides sculpturatus* embryo stained in same solution and for the same period of time as embryo shown in Figure 4D. (A’-C’) Counterstaining of embryos shown in (A-C) with Hoechst. Scale bars are 200 μm for (A, B) and 500 μm for (C). Click here for file

Additional file 4: Figure S3. Additional controls for chelicerate *cap-n-collar in situ* hybridization experiments. (A) Design of partially overlapping probes for *Po-cnc*. (B) Stage 14 embryo of *Phalangium opilio* in ventral view, stained with 739-bp anti-sense probe. (C) Stage 16 embryo of *Phalangium opilio* in ventral view, stained with 739-bp anti-sense probe. (D) Expression of *Cs-Antp*, a positive control for *Centruroides sculpturatus*. As in all chelicerates for which *Antp* expression data are available, *Cs-Antp* is expressed from the posterior part of the L4 segment to the posterior terminus. Dotted line indicates prosomal-mesosomal boundary. (D’) Counterstaining of embryos shown in (D) with Hoechst. ch, chelicera; ef, eye field; L, leg; lb, labrum; me: mesosoma; mt, metasoma; pp, pp, pedipalp; pr: prosoma. Scale bars are 200 μm for (B, C) and 500 μm for (D). Click here for file
