# Supplementary material for: Subdivision of arthropod cap-n-collar expression domains is restricted to Mandibulata
Source: EvoDevo. 2014 Jan 9;5:3. doi: 10.1186/2041-9139-5-3 (PMC3897911; doi:10.1186/2041-9139-5-3)
Supplement: Additional file 1: Figure S1. — Complete multiple sequence alignment of cap-n-collar orthologs. [file 2041-9139-5-3-S1.pdf]

Figure S1, Sharma et al.

Drosophila melanogaster	EESEIAEVLY	KQDVDLGFSL	DQEAIINASY	ASGNSAATNV	KSKPEDETKS	SDPSISESSG
Tribolium castaneum	MDMDLIEVLW	KQDVDLGFSL	D---------	----------	----------	---VANGKAD
Parhyale hawaiensis	----------	----------	----------	----------	----------	----------
Glomeris marginata	----------	----------	----------	----------	----------	----------
Phalangium opilio	----------	----------	----------	----------	----------	----------
Centruroides sculpturatus	----------	----------	----------	----------	----------	----------

Drosophila melanogaster	FKDTDVNAEN	EASAASVDDI	EKLKALEELQ	QDKDKNNENQ	LEDITNEWNG	IPFTIDNETG
Tribolium castaneum	KPDADLQNGS	PSSSISMEND	EIEKLKTLKA	INDDNIKEEP	ENELADPWAG	FNYTIDTETG
Parhyale hawaiensis	----------	----------	----------	----------	----------	----------
Glomeris marginata	----------	----------	----------	----------	----------	----------
Phalangium opilio	----------	----------	----------	----------	----------	----------
Centruroides sculpturatus	----------	----------	----------	----------	----------	----------

Drosophila melanogaster	EYIRLPLDEL	LNDVLKLSEF	PLQDDLSNDP	VASTSQAAAA	FNENQAQRIV	SETGEDLLSG
Tribolium castaneum	EYVV------	----------	-----KAEEL	SESLTGADCG	PSCDLPLGDL	SLPLPEFLLD
Parhyale hawaiensis	----------	----------	----------	----------	----------	----------
Glomeris marginata	----------	----------	----------	----------	----------	----------
Phalangium opilio	----------	----------	----------	----------	----------	----------
Centruroides sculpturatus	----------	----------	----------	----------	----------	----------

Drosophila melanogaster	EGISSKQNRN	EAKNKDNDPE	KADGDSFSVS	DFEELQNSVG	SPLFDLDEDA	KKELDEMLQS
Tribolium castaneum	EALRLVELDD	TPQEAINVKE	LENLAEGTNE	TEPSTSAPKA	SETPSSSKDS	DDDLSILTDM
Parhyale hawaiensis	----------	----------	----------	----------	----------	----------
Glomeris marginata	----------	----------	----------	----------	----------	----------
Phalangium opilio	----------	----------	----------	----------	----------	----------
Centruroides sculpturatus	----------	----------	----------	----------	----------	----------

Drosophila melanogaster	AVPSYHHPHP	HHGHPHAHPH	SHHHASMHHA	HAHHAAAAAA	AHQRAVQQAN	YGGGVGVGVG
Tribolium castaneum	IQTSQFHHPH	HRAFQGRMP-	----------	----------	----------	----------
Parhyale hawaiensis	----------	----------	----------	----------	----------	----------
Glomeris marginata	----------	----------	----------	----------	----------	----------
Phalangium opilio	----------	----------	----------	----------	----------	----------
Centruroides sculpturatus	----------	----------	----------	----------	----------	----------

Drosophila melanogaster	VGVGVGSGTG	SAFQRQPAAG	GFHHGHHQGR	MPRLNRSVSM	ERLQDFATYF	SPIPSMVGGV
Tribolium castaneum	----------	----------	----------	---FVRTVSM	EQRWQDLANL	LSLPSPGDTS
Parhyale hawaiensis	----------	----------	----------	----------	----------	----------
Glomeris marginata	----------	----------	----------	----------	----------	----------
Phalangium opilio	----------	----------	----------	----------	----------	----------
Centruroides sculpturatus	----------	----------	----------	----------	----------	----------

Drosophila melanogaster	SDMSPYPHHY	PGYSYQASPS	NGAPGTPGQH	GQYGSGANAT	LQPPPPPPPP	HHAAMLHHPN
Tribolium castaneum	GMPHPFSHHH	PLHNYSHPHA	HG--------	--------MA	YSPEATRGVL	LHNATLTPPM
Parhyale hawaiensis	----------	----------	----------	----------	----------	----------
Glomeris marginata	----------	----------	----------	----------	----------	----------
Phalangium opilio	----------	----------	----------	----------	----------	----------
Centruroides sculpturatus	----------	----------	----------	----------	----------	----------

Drosophila melanogaster	AALGDICPTG	QPHYGHNLGS	AVTSSMHLTN	SSHEADGAAA	AAAAYKVEHD	LMYYGNTSSD
Tribolium castaneum	GDINATVPYN	NIGGTNLGNA	VATSMNLTNS	SEPMGEPNSA	PHYKLEPSHD	MMYYQNSTTE
Parhyale hawaiensis	----------	----------	-----MFGKR	YFQDGAPPVP	PHHAGSNSSS	LV--------
Glomeris marginata	----------	----------	----------	----------	----------	----------
Phalangium opilio	----------	----------	----------	----------	----------	----------
Centruroides sculpturatus	----------	----------	----------	----------	----------	----------

Drosophila melanogaster	INQTDGFINS	IFTDEDLHLM	DMNESFCRMV	DNSTSNNSSV	LGLPSSGHVS	NGSGSSAQLG
Tribolium castaneum	LNQTDGFLSS	FLNDEDLQLM	DMAMNEGMYT	MRMLDSNNAV	SNLMSNGATG	TTTSLS----
Parhyale hawaiensis	----------	----------	----------	----------	----------	----------
Glomeris marginata	----------	----------	----------	----------	----------	----------
Phalangium opilio	----------	----------	----------	----------	----------	----------
Centruroides sculpturatus	----------	----------	----------	----------	----------	----------

```
Drosophila melanogaster  AGNPHGNQAN GASGGVGSMS GSAVGAGATG MTADLLASGG AGAQGGADRL DASSDSAVSS
Tribolium castaneum      ---------- ---------- ---------- ---------- ---RTDVERM DTSSDSAVSS
Parhyale hawaiensis      ---------- ---------- ---------- ---------- ---------- ----------
Glomeris marginata       ---------- ---------- ---------- ---------- ---------- ----------
Phalangium opilio        ---------- ---------- ---------- ---------- ---------- -----MTYQG
Centruroides sculpturatus ---------- ---------- ---------- ---------- ---------- ----------

Drosophila melanogaster  MGSERVPSLS DGEWGEGSDS AQDYHQGKYG GPYDFSYN-- ---------- --NNSRLSTA
Tribolium castaneum      MGSERVPSLS DGEWCDGGSD SGHTAGDHYV TDYHQTKYRP YDYSYTSRQH ASALAAASDA
Parhyale hawaiensis      ---------- ---------- ---------- ---------- ---------- ----------
Glomeris marginata       ---------- ---------- ---------- ---------- ---------- ----------
Phalangium opilio        IERNWNSRTN DGGTGTSSDS SASYSGMNYG ASYNEKKY-- ---------- ----------
Centruroides sculpturatus ---------- ---------- ---------- ---------- ---------- ----------

Drosophila melanogaster  TRQPPVAQKK HQLYGKRDPH KQTPSALPPT APPAAATAVQ SQSIKYEYDA GYASSGMASG
Tribolium castaneum      TRMPPVAQKK HQMFGKRYLQ EQGATSAVSH QPPVKYEYRD PTAAPYNSSQ ----------
Parhyale hawaiensis      ---------- ---------- ---GSSAAHY APAGAAAYLQ PTSAMDTTPA ----------
Glomeris marginata       ---------- ---------- ---------- ---------- ---------- ----------
Phalangium opilio        ---------- -------NPS DGNSNDYSSD SGCGKNNYQQ SDR------- ----------
Centruroides sculpturatus ---------- ---------- ---------- ---------- ---------- ----------

Drosophila melanogaster  GISEPGAMGP ALSKDYHHHQ PYGMGASGSA FSGDYTVRPS PRTSQDLVQL NHTYSLP---
Tribolium castaneum      ---PEGAVGP KPSE------ -MKYSCSMEF GRHSHLVRN- ---SLDHIQH NHTYHLP--T
Parhyale hawaiensis      ---PPPHM-P DPME------ -LKYSCSMDF -RGHHEVPP- ---SLDHIHH NHTYHMS--P
Glomeris marginata       ---------- ---------- ---------- ---------- ---------- ----------
Phalangium opilio        ---TPGTLYR SSTS------ -FLEGASNSY QKAETSTSR- ---SLNAFLH NHTYPTEYHN
Centruroides sculpturatus ---------- ---------- ---------- ---------- ---------- ----------

Drosophila melanogaster  QGSGSLPRPQ ARDKKPLVAT KTASKGASAG NSSSVGGNSS NLEEEHLTRD EKRARSLNIP
Tribolium castaneum      ESTGAMQRPV SRDK------ ---TKSKKG- ---------- --DEEHLTRD EKRARALNVP
Parhyale hawaiensis      EGPSGLPRPS QRDP------ ---SKHKAR- ---------K AEPERALTRD EKRARALNLP
Glomeris marginata       ---------- ---------- ---------- ---------- ---------- ----------
Phalangium opilio        EDSSNDSPPF DRDK------ ---KDNETKY RRKTTTESLD GSEGYYSSRD EKRARDLNLP
Centruroides sculpturatus --MCTKQKPV IRDK------ ---DYSDE-- ---------- ------ASRD ERRARMLKIP

Drosophila melanogaster  ISVPDIINLP MDEFNERLSK YDLSENQLSL IRDIRRRGKN KVAAQNCRKR KLDQILTLED
Tribolium castaneum      ITVDDIINLP MDEFNERLSK YDLSEPQLSL IRDIRRRGKN KVAAQNCRKR KLDQILSLAD
Parhyale hawaiensis      ISCDDIINLP MDEFNERISK YDLTEPQLSL IRDIRRRGKN KVAAQNCRKR KLDQILHLAE
Glomeris marginata       ---------- ------RLSK YDLTEPQLAL IRDIRRRGKN KVAAQNCRKR KLDQILVLAD
Phalangium opilio        LTAIEITTLP IDEFNERLSK YELTDEQLTL IRDIRRRGKN KIAAQNCRKR KMDQIFELQQ
Centruroides sculpturatus ISNEDIIHLP IDEFNERLSK YELKEEQLAL IKDIRRRGKN KVAAQNCRKR KIGQIMNLQE

Drosophila melanogaster  EVNAVVKRKT QLNQDRDHLE SERKRISNKF AMLHRHVFQY LRDPEGNP-C SPADYSLQQA
Tribolium castaneum      EVKDMRDRKM RLMNEHEYVT GECQRMKDKY QQLYRHVFQN LRDPDGNQ-Y SPYQYSLQTS
Parhyale hawaiensis      EVKVIQSRKS ELISQYEYLS GERLRIKHKF SLLYRHIFQH LRDSDGQP-Y SPHEYNLQQS
Glomeris marginata       EVTNMQSEKD QLLSEQQSMM AERQRLKDKF AQLYRHVFQT LRDPDGNP-Y SPYEYSLQQA
Phalangium opilio        TLEDLELERE KLTNEQSFVS RRRDFLQTKF NKLYRYICDM SPGPPDPPGY FPNQSSEGRR
Centruroides sculpturatus ELQSLLEEKA SLQAEQEQML S--------- ---YRR---- ---------- ----------

Drosophila melanogaster  ADGSVYLLPR EK-------- -----SEGNN TATAASNAVS SASGGSLNGH VPTQAPMHSH
Tribolium castaneum      ADGSILVVPR SN-------- -----STMTN PE-------- ---------- -HKEPPPQGH
Parhyale hawaiensis      ADGSVLLVPK SAPHIPLDPG LPPLPSNAND PEEDSSNS-- ---------R PPHQPPHQHH
Glomeris marginata       ADGNILLVPH NA-------- -----TNGME LD-------- ---------- -PSKGAKNKR
Phalangium opilio        PTNNFPMVNN ST-------- -----SFSNH HENAAENN-- ---------- -SHGGARVKR
Centruroides sculpturatus ---------- ---------- ---------- ---------- ---------- ----------

Drosophila melanogaster  QSHGMQ
Tribolium castaneum      KD----
Parhyale hawaiensis      TPH---
Glomeris marginata       RDDGKK
Phalangium opilio        KINKNN
Centruroides sculpturatus ------
```
