# Supplementary material for: Subdivision of arthropod cap-n-collar expression domains is restricted to Mandibulata
Source: EvoDevo. 2014 Jan 9;5:3. doi: 10.1186/2041-9139-5-3 (PMC3897911; doi:10.1186/2041-9139-5-3)
Supplement: Additional file 2: Table S1. — Gene-specific primers used for synthesizing cap-n-collar probes. [file 2041-9139-5-3-S2.docx]

**Table S1**

| **Species** | **Primer Name** | **Primer Sequence** | **Amplicon Length** |
| --- | --- | --- | --- |
| *Centruroides vittatus* | Cscu_cnc_for | TGAAGAAAGTATTCGTCATAATCACA | 393 bp |
|  | Cscu_cnc_rev | CGCCTGTATGAAAGCATTTG |  |
|  |  |  |  |
| *Parhyale hawaiensis* | Ph_cnc_T7_for | GGCCGCGGGACCCCATGGAGCTCAAATA | 575 bp |
|  | Ph_cnc_T7_rev | CCCGGGGCTGGTACCAGCAACACGCTAC |  |
|  |  |  |  |
| *Phalangium opilio* | Po_cnc_T7_for1 | GGCCGCGGGGAACTGGGACATCCTCTGA | 739 bp |
|  | Po_cnc_T7_rev | CCCGGGGCAAATATCCCGGAGGATCAGG |  |
|  |  |  |  |
|  | Po_cnc_T7_for2 | GGCCGCGGCGCGTTCCTTCATAATCACA | 488 bp |
|  | Po_cnc_T7_rev | CCCGGGGCAAATATCCCGGAGGATCAGG |  |
