# Supplementary figures and images for: Subdivision of arthropod cap-n-collar expression domains is restricted to Mandibulata
Source: EvoDevo. 2014 Jan 9;5:3. doi: 10.1186/2041-9139-5-3 (PMC3897911; doi:10.1186/2041-9139-5-3)

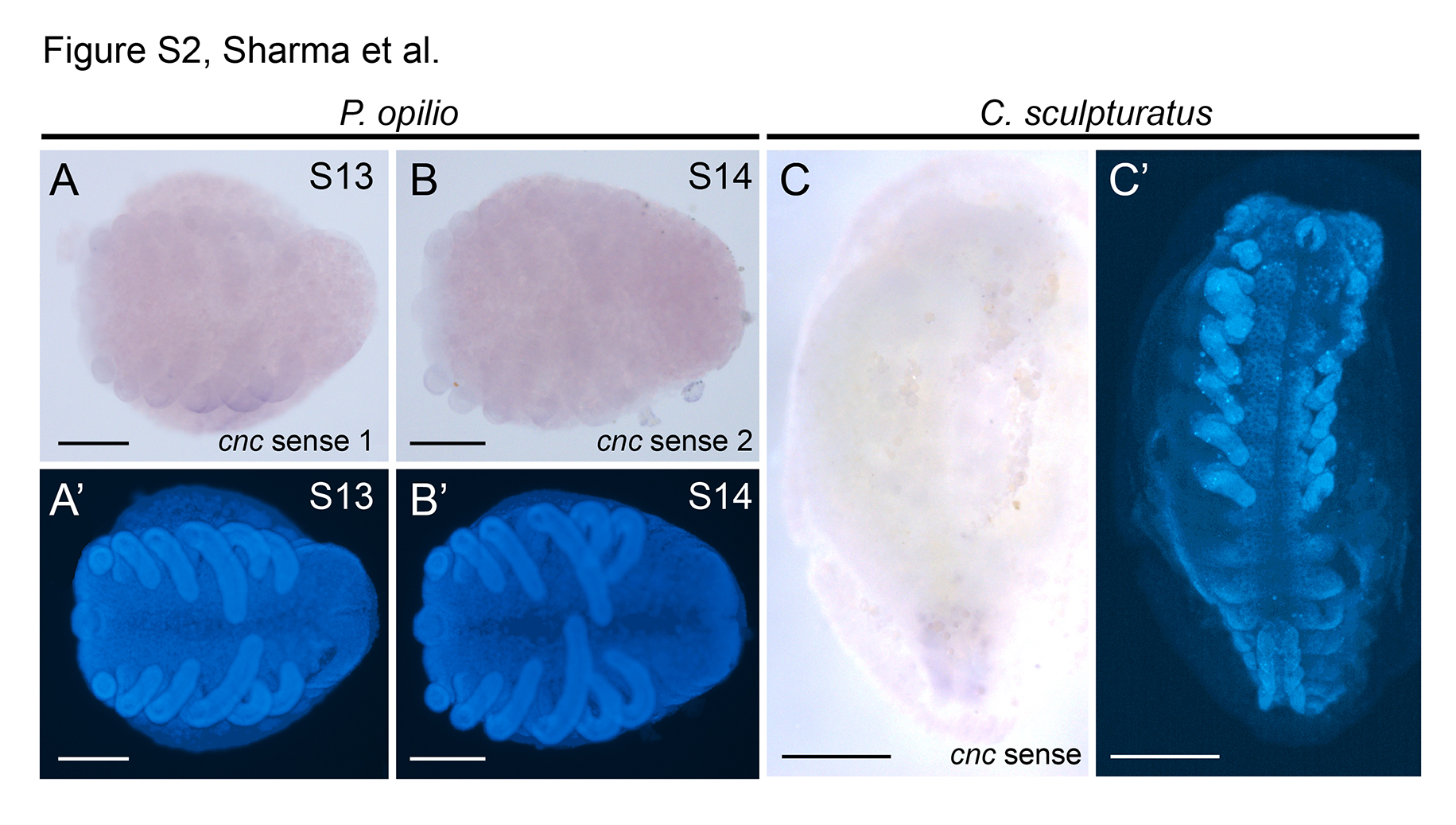

Supplement: Additional file 3: Figure S2. — Chelicerate cap-n-collar sense probes. (A) Stage 13 embryo of Phalangium opilio stained with 488-bp sense probe in same solution and for the same period of time as embryos shown in Figure 4A-C. (B) Stage 14 embryo of Phalangium opilio stained with 739-bp sense probe in same solution and for the same period of time as embryos shown in Additional file 4: Figure S3B-C. (C) Centruroides sculpturatus embryo stained in same solution and for the same period of time as embryo shown in Figure 4D. (A’-C’) Counterstaining of embryos shown in (A-C) with Hoechst. Scale bars are 200 μm for (A, B) and 500 μm for (C). [file 2041-9139-5-3-S3.jpeg]

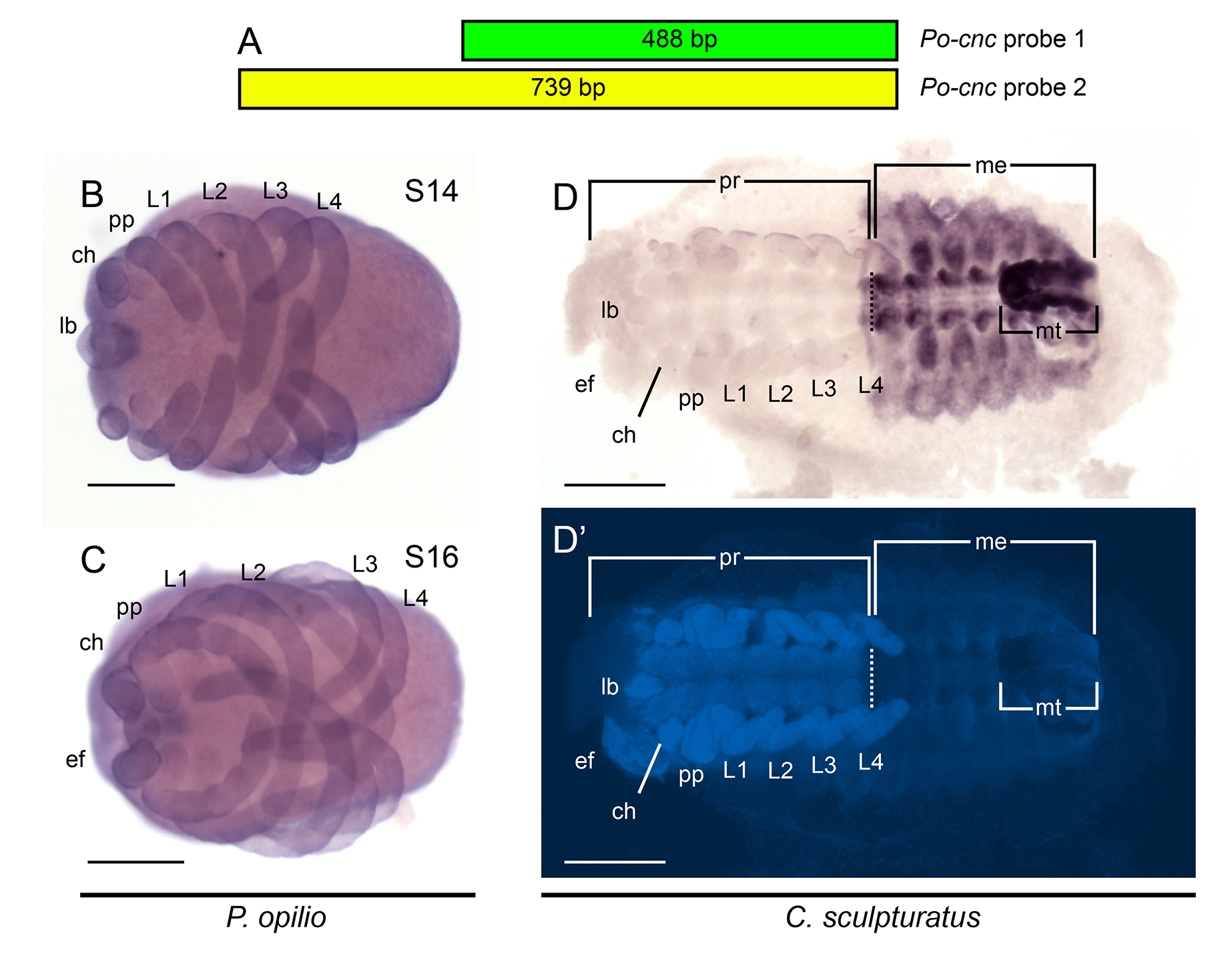

Supplement: Additional file 4: Figure S3. — Additional controls for chelicerate cap-n-collar in situ hybridization experiments. (A) Design of partially overlapping probes for Po-cnc. (B) Stage 14 embryo of Phalangium opilio in ventral view, stained with 739-bp anti-sense probe. (C) Stage 16 embryo of Phalangium opilio in ventral view, stained with 739-bp anti-sense probe. (D) Expression of Cs-Antp, a positive control for Centruroides sculpturatus. As in all chelicerates for which Antp expression data are available, Cs-Antp is expressed from the posterior part of the L4 segment to the posterior terminus. Dotted line indicates prosomal-mesosomal boundary. (D’) Counterstaining of embryos shown in (D) with Hoechst. ch, chelicera; ef, eye field; L, leg; lb, labrum; me: mesosoma; mt, metasoma; pp, pp, pedipalp; pr: prosoma. Scale bars are 200 μm for (B, C) and 500 μm for (D). [file 2041-9139-5-3-S4.jpeg]
